# Association between CFH Y402H Polymorphism and Age Related Macular Degeneration in North Indian Cohort

**DOI:** 10.1371/journal.pone.0070193

**Published:** 2013-07-29

**Authors:** Neel Kamal Sharma, Amod Gupta, Sudesh Prabhakar, Ramandeep Singh, Suresh Kumar Sharma, Wei Chen, Akshay Anand

**Affiliations:** 1 Department of Neurology, Post Graduate Institute of Medical Education and Research (PGIMER), Chandigarh, India; 2 Department of Ophthalmology, Post Graduate Institute of Medical Education and Research (PGIMER), Chandigarh, India; 3 Department of Statistics, Panjab University, Chandigarh, India; 4 Division of Pulmonary Medicine, Allergy and Immunology, Children’s Hospital of Pittsburgh of UPMC, Pittsburgh, Pennsylvania, United States of America; Sanjay Gandhi Medical Institute, India

## Abstract

The purpose of the study was to determine serum complement factor H (CFH) levels in patients of age related macular degeneration (AMD) and examine its association with CFH Y402H polymorphism. 115 AMD patients and 61 normal controls were recruited in this study. The single nucleotide polymorphism was assayed by real time PCR and serum CFH levels were measured by ELISA and standardized to total serum protein. Chi-square test was applied to polymorphism analysis while Mann Whitney U-statistic for CFH-levels. Mendelian randomization approach was used for determining causal relationship. The genotype frequency differed between the AMD patients (TT- 18.3%, TC-41.3% and CC-40.4%) and controls (TT-76.3%, TC-13.6%, and CC-10.1%) (p = 0001). The frequency of alleles was also significantly different when AMD (T-39% and C-61%) was compared to controls (T-83% and C-17%) (p = 0.0001). Level of serum CFH was significantly lower in AMD patients as compared to normal controls (p = 0.001). Our data showed that the CFH Y402H polymorphism is a risk factor for AMD in the North Indian population. Mendelian randomization approach revealed that CFH Y402H polymorphism affects AMD risk through the modification of CFH serum levels.

## Introduction

AMD is a progressive disease of the retina and a leading cause of irreversible visual impairment [1], [2]. AMD has two stages: early stage and advanced stage. In the early phase of disease there is presence of soft drusen with hyperpigmented and pigmented area. With time a few of early AMD may progress to advanced stage [1]. First is the dry AMD, which is marked by drusen or depigmentation caused by products of the photoreceptors and retinal pigment epithelium (RPE). The next phase of disease is called wet AMD because it is due to the growth of new abnormal blood vessels under the neurosensory retina and RPE, which results in subretinal bleeding and consequent scar formation. Both types of AMD may lead to central vision loss but 90% vision loss is known to be due to wet AMD. Fewer than 1% of the affected patients are under the age of 65 years, which increases with age, to 9% over 65 years and up to 30% over 70 years [3]. Therefore, the increasing population of elderly individuals impact health economics of every nation. The prevalence of AMD in India ranges from 1.84–2.7% [4].

AMD results from both environmental and genetic factors, even though its actual etiology remains unclear. CFH single nucleotide polymorphisms [SNPs] have been reported as the most important genetic risk factors for AMD pathogenesis.

Some independent studies have suggested that Y402H polymorphism in CFH gene plays an important role in determining AMD susceptibility (Y402H has a TrC substitution in exon 9 at 1277 nucleotide, which results in a tyrosine to histidine change) [5]–[7].

Another study from India has also reported significant association of Y402H among AMD patients (p = 1.19×10^–7^). They showed that persons homozygous for CC had a significantly higher risk (p = 0.0001) of AMD than heterozygous genotype [8].

CFH has been reported to be present in human and mouse ocular tissues such as RPE and choroid and is associated with drusen in AMD patients [9], [10]. AMD is associated with complement dysregulation or activation of the spontaneously initiated alternative complement pathway leading to local inflammation, which is involved in pathogenesis of disease. CFH is known to be involved in maintaining homeostasis of complement system and any alteration in this system either in the form of altered functions of CFH variants or CFH expression could lead to activation of complement systems which triggers further events leading to cell damage of the RPE cells, formation of drusen and visual loss [11]. Complement components C3a and C5a are prominently involved in the AMD [12]. C3a deposition and C5a release after complement activation are inhibited by Complement factor H, any defect in CFH induces increased production of C3a and C5a frequently seen in AMD autopsies [13] thus confirming a local role of inflammation and complement in the pathogenesis of AMD. We hypothesized that a mutation in CFH could affect the CFH protein levels.

The purpose of this study was to determine the frequencies of the CFH Y402H variants and the levels of serum CFH in AMD patients and normal controls in the north Indian population, a study which has not been undertaken earlier. In this study, we applied Mendelian randomization approach to test whether CFH polymorphism, CFH levels and other confounders have any role in the etiology of AMD.

## Materials and Methods

### Patients and Control Individuals

Two independent groups of North Indian population including patients of AMD and controls were recruited in the study through the retina clinic, Department of Ophthalmology, Post Graduate Institute of Medical Education and Research (PGIMER) Chandigarh, India. The study was approved by institutional ethics review committee of PGIMER, Chandigarh (No. Micro/10/1411). Patients were enrolled in the study based on approved inclusion and exclusion criteria after written informed consent was obtained. We included 176 case-control samples consisting of 115 AMD patients along with 61 genetically unrelated healthy controls. We have excluded those cases in which any demographic detail was lacking.

Only those AMD patients were recruited who fulfilled the inclusion criteria such as those with an age 50 years or more with a diagnosis of AMD defined by dry and/or choroidal neovascularization with five large drusen or more [14]. The controls were of age 50 years or older with no drusen and absence of other diagnostic criteria defined for AMD.

All patients and controls were examined by a retina surgeon for visual acuity measurement, and dilated fundus examination. All patients underwent fluorescein fundus angiography. AMD diagnosis was based on ophthalmoscopic and fluorescein angiographic findings.

A standardized risk factor questionnaire was used by a trained interviewer to interview all the subjects [15]–[17]. Demographic information such as alcohol intake, cigarette smoking, food habits and comorbidity were included in a questionnaire. Smokers were defined as those having smoked at least three cigarettes per day or 54 boxes for at least 6 months. Non vegetarian patients were defined as those having chicken, meat or fish for at least 6 months. Information about alcohol use for at least 6 months was also collected. Co-morbidities were determined based on the participant’s answers to whether a physician had ever informed them for diagnosis of any main neurological, cardiovascular or metabolic illness.

### Sample Collection

Blood samples were collected from all subjects. Serum was separated from 4.0 ml of blood by using serum separator tubes (BD Biosciences, USA). Genomic DNA was extracted from the peripheral venous blood using a commercial kit (QIAGEN, Germany and INVITROGEN, USA) according to the manufacturer’s protocol. The samples were coded, labeled and stored in −80°C freezer until assayed.

### Protein Analyses

The quantification of serum total protein was done using Bradford assay in order to standardized CFH levels estimated from ELISA. The CFH protein levels were analysed using commercially available enzyme linked immunosorbant assay (ELISA; Cusabiotech; Catalog no. CSB-E08931h) according to manufacturer’s procedure and the absorbance was taken at 450 nm by 680XR Microplate reader (Biorad, Hercules, USA). This assay recognizes recombinant and natural human CFH with detection range of 15.6 µg/ml-1000 µg/ml. All the samples were analysed in duplicates. The standard curve for CFH estimation was done by linear regression analysis. All the values were standardized with total serum protein.

### Genotyping

The SNP (rs1061170) employed for analysis in our study was previously documented in other ethnic populations for involvement with AMD and was chosen based on its functional significance. It is defined as Y402H. The rs1061170 (T) allele encodes the more common Tyr (Y), while the generally rarer rs1061170(C) encodes the His (H). The SNP (rs1061170) assay was done by using Real time PCR (Applied Biosystems Inc., Foster city, CA) using published TaqMan® SNP Genotyping Assays (7). Real time PCR was carried out for 20.0 µl volume containing 10 ul master mix, 5 ul Assay (Applied Biosystems) and 20 ng DNA was added to make the volume 20.0 µl. TaqMan® SNP Genotyping Assays (Applied Biosystems) was used to carry out all reactions according to manufacturer’s recommendations. Two reporter dyes FAM and VIC were used to label the Allele 1 and 2 probes and 5′ Nuclease Assay was carried out. PCR mix without DNA was used as negative control. StepOne™ v 2.0 software (Applied Biosystems Inc., Foster city, CA) was used to perform the genotype calling and Sequence Detection System (SDS) Software was used to import the fluorescence measurements made during the plate read to plot fluorescence (Rn) values after PCR amplification (2).

### Statistical Analysis

After taking the log of CFH ELISA values it was observed from Normal Quantile plot (Q-Q plot) that the data was approximately normally distributed. t-test was therefore, applied for comparing the two groups. For comparing more than two groups, One-way analysis of variance (ANOVA) followed by post-hoc was applied for multiple comparisons. The p≤0.05 was considered significant. The measure R^2^ (Coefficient of determination) was used to determine the goodness of standard curve fit for ELISA and total protein. The linear and quadratic regressions with R^2^>0.80 were considered to be a good fit. The genotypes were stratified for homozygosity and heterozygosity of the respective allelic variant. Association between various study groups was done by using Pearson’s Chi-square test. Odds ratios (ORs) with 95% CI and genotypic associations were estimated by binary logistic regression. All statistical analysis such as linear regression, quadratic fit and test of significance were performed with statistical package and service solutions (SPSS; IBM SPSS Statistics 20.0, Chicago, Illinois, USA) 20.0 software. Mendelian randomization (MR) approach was used to investigate the CFH causal pathway in our study.

## Results

Summary statistics of all important variables are reported in Table 1.

**Table 1:** Demographic characteristics of Controls and AMD patients.

Variables	AMD	Controls
Number	115	61
Age	64.97±7.1	60.38±13.2
Duration of disease^¥^	23±2.6 (M)	–
Wet AMD	84 (73.04%)	–
Minimal Classic	7 (11.9%)	–
Predominant Classic	16 (27.1%)	–
Occult	36 (61.0%)	–
Familial Cases	10 (8.7%)	–
Bevacizumab Treated	55 (65.5%)	–
Smokers	50 (43.5%)	11 (20%)
Alcohol User	37 (32.2%)	17 (30.9%)
Vegetarian	61 (53%)	31 (56.4%)
Male	75 (65.2%)	40 (65.6%)

Clinical and demographic details of subjects. AMD, age related macular degeneration; M, Months; Age, Age of onset; Values are mean ± SD or (percentage), ¥ Duration of disease is the interval between appearance of first symptom of AMD and collection of sample. AMD subjects were asked to provide all clinical and demographic details at the age of disease-onset.

### Single Nucleotide Polymorphism

We analyzed one polymorphism in the CFH gene by real time PCR. Genotype and allele frequencies of CFH have been listed in Tables 2. There was a significant difference between the homozygous genotype frequency for allele T, homozygous genotype frequency for allele C and heterozygous genotype frequency between AMD patients and normal controls. The CC and TC genotypes were more frequent in AMD patients than to controls (OR = 16.5, CI = 6.05–44.96, p = 0.0001, and OR = 12.65, CI = 5.05–31.69, p = 0.0001 respectively, Table 2). The C allele was more frequent in AMD cases than controls (OR = 7.6, CI = 4.4–13.3, p = 0.0001, Table 2). There was no significant difference in the genotype and allele frequencies between wet and dry AMD patients (Tables 2). Logistic regression analysis for eating habits, smoking and presence of comorbidity revealed that the TC genotype was more frequent in vegetarian AMD patients (OR = 4.22, CI = 1.35–13.15, p = 0.012, Table 3) and AMD patients with comorbodities (OR = 3.68, CI = 1.145–11.83, p = 0.028, Table 3). The difference was not significant when compared for bevacizumab treatment, the number of eyes affected and between wet AMD patients ie minimally classic, predominantly classic and occult (data not shown).

**Table 2:** Genotype and allele frequency of CFH rs1061170 by Logistic Regression analysis.

			Unadjusted p value			Multivariate analysis, adjusted for age, gender, food habits, smoking and comorbidity		
**Genotype**	**Number (frequency)**		**OR**	**95% CI**	**p Value**	**OR**	**95% CI**	**P Value**
**CFH rs1061170**								
	**AMD**	**Controls**						
TT	20 (.183)	45 (.763)	Reference			Reference		
TC	45 (.413)	8 (.136)	12.65	5.05–31.69	0.0001	1.960	0.393–3.526	0.014
CC	44 (.404)	6 (.101)	16.5	6.05–44.96	0.0001	*	*	*
	**Wet AMD**	**Dry AMD**						
TT	16 (.20)	4 (.138)	Reference			Reference		
TC	33 (.412)	12 (.414)	0.69	0.191–2.471	0.566	1.447	0.897–3.791	0.226
CC	31 (.388)	13 (.448)	0.60	0.167–2.129	0.426	0.400	0.049–3.289	0.394
**Allele**	**Number (frequency)**		**OR**	**95% CI**	**p Value**			
	**AMD**	**Controls**						
T	85 (.39)	98 (.83)	Reference					
C	133 (.61)	20 (.17)	7.6	4.4–13.3	0.0001	-	-	-
	Wet AMD	Dry AMD						
T	65 (.41)	20 (.34)	Reference					
C	95 (.59)	38 (.66)	0.77	0.41–1.43	0.41	-	-	-

* The value could not be complied because of the equal frequencies.

**Table 3:** Logistic regression of CFH rs1061170 and AMD stratified by food habits, smoking and comorbidities.

			Unadjusted p value			Multivariate analysis, adjusted for age and sex		
Genotype	Number (frequency)		OR	95%CI	p-value	OR	95%CI	p-value
**CFH rs1061170**								
	**Vegetarian AMD**	**Non Vegetarian AMD**						
TT	6 (0.10)	14 (0.28)	Reference			Reference		
TC	29 (0.50)	16 (0.31)	4.22	1.35–13.15	0.012	0.404	0.113–1.445	0.164
CC	23 (0.40)	21 (0.41)	2.55	0.83–7.86	0.102	2.579	0.589–11.29	0.209
	**AMD Smokers**	**AMD Non Smokers**						
TT	9 (0.19)	11 (0.18)	Reference					
TC	22 (0.47)	23 (0.37)	1.16	0.40–3.36	0.772	1.00	0.271–3.694	1.00
CC	16 (0.34)	28 (0.45)	0.69	0.23–2.04	0.512	0.412	0.70–2.42	0.327
	**AMD with Comorbodities**	**AMD without Comorbodities**						
TT	11 (0.14)	9 (0.32)	Reference			Reference		
TC	36 (0.46)	8 (0.29)	3.68	1.145–11.83	0.028	0.083	0.08–0.898	0.040
CC	32 (0.40)	11 (0.39)	2.38	0.779–7.265	0.127	1.836	0.462–7.29	0.388

A logistic regression analysis was performed to analyze the association between the SNP and other risk factors with AMD simultaneously. We analyzed age, sex, food habits smoking and comorbidity as risk factors which have been shown to be associated with AMD previously. When multiple logistic regression analysis was carried out with adjustment for age, sex, food habits, smoking and comorbidity, we found that TC genotype was at higher frequency in AMD patients than controls (p = 0.014, Table 2). Sex and age adjustment for AMD patients with comorbidity also showed higher frequency of the TC genotype than in AMD patients without comorbidity (p = 0.040, Table 3).

### Serum Levels of CFH are Decreased in AMD Patients

**Figure 1:**
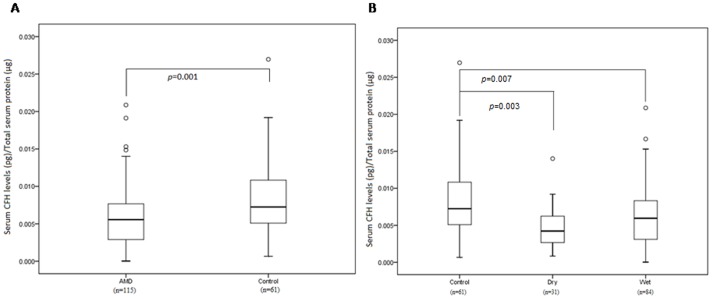
A) Serum levels of CFH in AMD and normal controls. B) Serum levels of CFH in Controls, Dry and Wet AMD. Boxes include values from first quartile (25th percentile) to third quartile (75th percentile). Outliers and extreme values are shown in circles and asterisk respectively. Levels of CFH were standardized to total protein. AMD, Age Related Macular Degeneration; CFH, Complement Factor H; pg, picogram; µg, microgram.

We investigated the serum CFH levels in AMD and controls. We also examined the correlation between CFH genotype and protein expression in serum. The CFH levels in AMD were significantly lower than in controls (Figure 1A, Table 4, p = 0.001). However, we did not find significant difference in the CFH serum levels between wet and dry AMD patients, but both patients groups had significantly lower levels than controls (Figure 1B, p = 0.007, 0.003 respectively, Table 4). To estimate the predictive value of CFH, serum levels of CFH were again segregated into minimal classic, predominantly classic and occult AMD. The difference was not significant between the wet AMD subgroups (Table 4). We did not find any significant difference when the ELISA levels were compared to other parameters like smoking, alcohol, eating habit and bevacizumab treatment (Table 4). We did not find any significant correlation between CFH genotype and protein expression in serum.

**Table 4:** Log CFH Serum levels according to AMD and control subtypes (Comparison using t-Statistic).

Subjects CFH	Mean	t-Value	p-value
AMD	−5.37		
Control	−4.94	−3.27	0.001
Dry	−5.49		
Wet	−5.32	−0.85	0.400
Bevacizumab treated	−5.34		
Not treated	−5.29	0.216	0.830
Minimal Classic	−5.50		
Predominant Classic	−5.59	0.124	0.871
Occult	−5.37	−0.327	0.806
Alcohol consumption	−5.46		
No Alcohol consumption	−5.32	0.670	0.50
Smokers	−5.39		
Non Smokers	−5.35	0.244	0.807
Vegetarian	−5.45		
Non Vegetarian	−5.28	0.98	0.331
Without comorbidities	−5.31		
With comorbidities	−5.39	0.43	0.670

### Mendelian Randomization Approach

**Figure 2:**
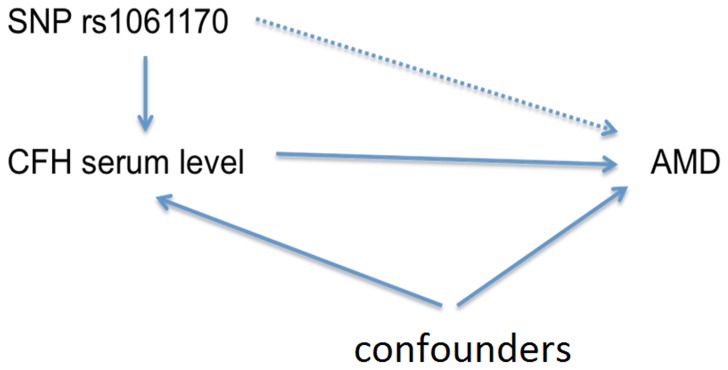
Mendelian randomization approach.

We used the Mendelian randomization (MR) approach [18], [19] to investigate the potential causal pathway by including SNP, CFH serum levels, and AMD with other risk factors (food habit, comorbidity, and smoking). CFH rs1061170 was analyzed as an instrumental variable for CFH serum level. We found that allele C increased the risk of AMD and lower CFH serum level was observed in AMD patients. Allele C reduces the CFH serum level, but SNP was also associated with food habit and comorbidity. Therefore, the causal effect of CFH serum on the risk of AMD may require further studies. We illustrate this approach in Figure 2.

## Discussion

The Y402H polymorphism in CFH is a major risk factor for AMD [6], [7]. The non-synonymous variant (T-C) results in tyrosine to histidine transformation at codon 402 of this loci. Several studies have established an association of the CFH gene, which is an inhibitor of the alternative complement activation pathway to be responsible for AMD. Association of the Y402H (rs1061170) variant of CFH with AMD has been described in several populations worldwide [6], [20], with TC and CC genotype being approximately 2.5 and 6 times extra likely to have AMD than patients having TT genotype [21], and this was later confirmed in Italian [22], French [7], British [6], Russian [14] and Icelandic [20] populations. However, it appears to be less common in Chinese [23], and is absent in Japanese [24], [25] but no such study has been conducted in the homogeneous population from Northern India.

This study was therefore conducted to determine the prevalence of CFH polymorphism and to test whether differences in levels of serum CFH exist between Indian patients with AMD and healthy controls. We report significantly lower serum CFH levels in AMD patients as compared to controls and Y402H variant of CFH to be associated with AMD in this population. Homozygous CC and heterozygous TC genotypes were more frequent among AMD patients than controls. Moreover, the CC and TC genotypes conferred OR for AMD of 16.5 and 12.6, respectively. CFH is involved in the inflammatory response of the innate immune system. Low levels of CFH in North Indian population is consistent with other reports. Dhillon et al showed that the prevalence of factor H autoantibodies decreased in AMD patients as compared with normal controls [26]. Some investigators have shown that reduced serum CFH is associated with obesity, hypertension and smoking which are known risk factors for AMD [27], [28]. In a recent study, Silva et al observed significant differences in the plasma levels of the alternative pathway proteins i.e. Factor D (FD) and Factor I (FI) between the AMD patients and control. They showed significantly lower FD plasma levels and higher FI levels in AMD patients and also identified a significant decrease in CFH plasma levels in AMD females patients in relation to normal females [29].

Several studies have previously examined the role of CFH Y402H polymorphism in the AMD subtypes such as geographic atrophy (GA) or choroidal neovascularization (CNV). The weakly regulated complement cascade, due to CFH polymorphism, might enhance cellular damage, ultimately leading to atrophy or neovascular response [30]. In the patients investigated the Y402H polymorphism was not predictive for either of these AMD phenotypes. This supports the concept that it could be involved in both dry and wet AMD variants [31]. It is pertinent to note that conflicting results exist where such associations have been investigated wherein some groups have suggested that neovascular AMD to be at a higher risk of this genotype variant [32], [33] while others noting that atrophic AMD represents a higher risk of this polymorphism [34], [35], however, there are many others who have reported it to bear no variation with AMD phenotype [17]. Our results are not consistent with those that suggest association with neovascular or dry AMD.

There are certain reports indicating increased risk for each successive stage of AMD associated with the CFH polymorphism [36]. Our findings do not show any difference between minimal classic, predominantly classic and occult AMD in the association with the CFH Y402H genotype. Interestingly, our findings also raise questions about the role of eating habits and other co-morbidities on individual genotype. We, however, note that AMD has previously been reported to be associated with other diseases such as stroke and depression [37], [38]. Vegetarian diet and existence of co-morbidities in AMD patients seemed to suggest a non redundant association with the TC genotype and the risk of developing AMD with OR = 4.22 and 3.68, respectively. The importance of this association is unclear due to limited data. However, those on vegetarian diet including those not consuming fish, may be deficient in a essential nutrients – especially docosahexaenoic acid (DHA) and eicosapentaenoic acid (EPA) the long-chain omega-3 fatty acids. Alphalinolenic acid (ALA) is an omega-3 fat and is the precursor of the longer chain omega 3 fats EPA and DHA, i.e. ALA in the body can form EPA and to a lesser extent DHA. Some fish and seafood are the major dietary sources of these fatty acids. As a result, vegetarian diets provides little DHA and EPA. Kornsteiner et al showed that vegetarians are left with less omega-3 levels [39]. In addition, ALA, DHA, and EPA are particularly important for the prevention of AMD [40]. Some studies have reported that fish consumption and omega-3 fatty acid intake reduces the risk of AMD [41], [42]. However, some studies suggest an inverse relation between regular dietary intake of DHA, EPA, fish and risks of advanced AMD [43], [44]. Recent unpublished reports from Punjab, India have also shown correlation between excess use of pesticides in agricultural crops and incidence of cancer and other degenerative disorders (http://health.india.com/diseases-conditions/are-the-farmers-in-punjab-paying-a-price-for-the-green-revolution/).

Using a Mendelian randomization approach, our results show strong evidence that CFH serum levels are causal to AMD, which strengthens the study. This implies: Allele C increases the risk of AMD; lower CFH serum level is observed in AMD patients; Allele C reduces the CFH serum level. This evidence strengthens the argument that increasing CFH serum level might lower the risk of AMD. SNP was also found to be associated with food habit and comorbidity. Therefore, the correlation of CFH serum on the risk of AMD may require further studies. The key limitation of the study was the lack of local tissue.

### Conclusion

Our study demonstrated that the CFH Y402H polymorphism with a higher frequency of the allele may affect the CFH serum levels resulting in AMD.
